# Deficiencies of the EU Medical Device Regulation when applying its own rules

**DOI:** 10.3389/fmed.2025.1732399

**Published:** 2026-01-21

**Authors:** Martin Haimerl, Michael D’Agosto, Mike Fornefett, Massimo Kubon, Thomas Schiepp

**Affiliations:** 1 Faculty Engineering & Technology, Furtwangen University, Furtwangen, Germany; 2 Innovation and Research Center Tuttlingen of Furtwangen University, Furtwangen University, Tuttlingen, Germany

**Keywords:** EU Medical Device Regulation, good regulatory practice, medical devices, product development (PD) process, regulatory systems evaluation, risk-based approach

## Abstract

**Introduction:**

The EU Medical Device Regulation (MDR) was developed to improve the safety of medical devices as well as the smooth functioning of the market in this field. Since it entered into force in 2017, there have been continuous debates about the actual success thereof. However, no consequent analysis was provided until now. In this paper, we contribute to this endeavor. We analyzed whether the MDR meets appropriate standards that it requires for the development of medical products. In other words, we raised the question of whether the MDR could be placed on the market if we were to apply the rules of the MDR to itself.

**Methods:**

For this purpose, the analysis was based on the MDR and its Recitals, Articles, and Annexes, as well as the components implementing the regulatory system based on it. We checked whether basic principles for product development and the setup of regulatory systems in the field of medical devices are fulfilled. We asked whether basic development steps for the MDR have been realized appropriately, according to these principles and standards.

**Results:**

The analysis showed that the MDR contains substantial deficiencies regarding a consequent implementation of product development standards. For example, this applies to core principles like transparency, clarity, and traceability of requirements, appropriate implementation of risk management and validation steps, or realization of governance structures. According to our findings, the MDR would fail when we applied its own rules. In particular, central goals of the MDR like the smooth functioning of the market and the safety of the regulatory system were not addressed consequently.

**Discussion:**

According to the high impact the MDR has on the medical device sector but also on the healthcare system in general, our analysis motivates improvements of the MDR-based regulatory system that take these deficiencies into account. This paper provides basic insights into the application of basic principles regarding the implementation of regulatory systems. This should be complemented by further evaluation steps regarding the actual performance of the MDR during its operational phase. Based on this, consequent steps for the actual improvement should be derived to finally achieve a high-quality regulatory system.

## 1 Introduction

A well-functioning regulatory system for medical devices is a key element for achieving high performance in the associated health care system. In Europe, the medical device regulation (MDR, Regulation (EU) 2017/745 of the European Parliament and of the Council of 5 April 2017 on medical devices) (1) was established to achieve this goal. The MDR was developed and introduced after the appearance of a set of issues with the previously applicable Medical Device Directive (MDD) (2). In particular, safety issues regarding breast implants as well as metal-on-metal hip implants were a driving force for replacing the MDD with the MDR (3–7). Based on these experiences, further improvement in product safety was a major goal of the MDR, besides other objectives like the harmonization of the rules on medical devices and the integration of the previously existing directives into one EU-wide more consistent regulatory system (1, 8, 9). The development of the MDR already started in 2008, shortly after the mentioned issues were identified (3). However, it lasted until 2017 before the MDR entered into force. Since then, numerous discussions have taken place regarding the proper setup and implementation of the MDR (3). Based on this, a series of amendments were made, e.g., regarding the shift of timelines for the execution of particular requirements. Additionally, the availability of important infrastructure was substantially delayed. In particular, this applies to the availability of notified bodies in the initial phase of the MDR or the Eudamed database, whose full functionality could not be provided in 2025, even 8 years after the MDR was published (3, 5, 10, 11).

Thus, it should be asked whether the MDR was successful and achieved the intended goals. In Article 121, the MDR itself includes a requirement that an evaluation needs to be performed in order to “assess the application of this Regulation and produce an evaluation report on the progress towards achievement of the objectives contained herein including an assessment of the resources required to implement this Regulation” (1). The MDR set the timeline for this evaluation to 2027, i.e., 10 years after the entering into force. However, the discussions about potential deficiencies of the MDR resulted in an earlier timeline for this step. The EU Commission initiated a targeted evaluation of the MDR and also the *In Vitro* Diagnostics Regulation (IVDR) (12) that included a public consultation in the time frame from Dec 12, 2024 until Mar 21, 2025 (13). Finally, 584 statements (duplicated entries not removed) were provided in the public consultation from a broad range of stakeholders, including industry, research, authorities, trade unions, non-governmental organizations, and private EU citizens. In particular, the feedback contained reports about particular issues with the MDR, concrete options for improvements, as well as general remarks about the performance of the MDR (13). Additionally, a series of studies and position statements from authorities, trade unions, non-governmental organizations, and academic institutions emphasized deficiencies in the setup and implementation of the MDR (4, 6, 7, 10, 11, 14–17).

This underpins the need for a thorough evaluation of the MDR regarding its performance and effectiveness. This does not only include the MDR itself but also the regulatory system built around it. Thus, also tools and infrastructure that are important for the implementation of the MDR need to be considered. The same applies to responsible actors as defined in the MDR as well as the corresponding governance structures. In this paper, we performed a systematic analysis in this direction. We did not provide more study data about the actual performance and potential issues of the MDR, since this already was addressed in publications like Huusko et al. (4), Nüssler (6), Kearney and McDermott (7), Shatrov and Blankart (14), Svempe (15), and Deutsche Industrie und Handelskammer, MedicalMountains, Spectaris (16). Instead, we provide an analysis about the realization of the MDR from a product development perspective. Since the MDR has to be considered as a key element for the performance and effectiveness of the health care system in EU member states, the MDR should set up and pursue appropriate goals to achieve this. In this sense, the MDR can be considered as a product that needs to define and implement appropriate requirements – across the complete development chain including specification, implementation, verification and validation, as well as post market surveillance and governance processes. For good reasons, the MDR sets high standards on medical devices to ensure appropriate safety and performance of these devices. The MDR itself should align to these standards in order to successfully pursue its own mission.

In this paper, we investigated whether the MDR itself and the regulatory system built on it are able to meet such standards. This refers to the question of whether the MDR could be placed on the market if its own rules were applied. Starting from an analysis of appropriate goals, we systematically analyzed how consequent such requirements were defined and implemented in the MDR. In this regard, we applied basic principles for product development but also for the setup of regulatory systems in the field of medical devices. Our study was directly based on the analysis of the MDR itself, including its Recitals, Articles, and Annexes. Where needed, further references were utilized to substantiate the findings. We did not provide a comprehensive analysis of all parts of the MDR but highlighted the consequent implementation and impact of important components in an exemplary way. In other words, we focused on the analysis of prominent example to showcase the deficiencies of the MDR. A comprehensive analysis would have exceeded the scope of the paper. These particular aspects are intended to demonstrate major deficiencies in the current state of the MDR and also potential improvements. Our paper does not include a dedicated analysis of the IVDR. But due to the similarities between the MDR and IVDR, many of the analysis steps also apply to the IVDR.

## 2 Materials and methods

In this paper, we investigated whether the MDR itself was realized according to high-quality standards that are comparable to established requirements for product development and regulatory systems in general. In other words, we asked whether the MDR provides a similar level of sophistication as it requires from medical devices. In particular, this means that the MDR and the regulatory system built on it need to pursue a clear definition of goals and requirements as well as a consequent implementation of them. Following quality standards, it should also establish appropriate processes to guarantee a high level of performance across its lifecycle. Additionally, it should provide sufficient evidence that the goals and requirements are met. In summary, we investigated whether the following basic steps of a standard product lifecycle are pursued in the MDR appropriately.

S1 Definition of basic goals.
S2 Requirements engineering and specification.
S3 Implementation.
S4 Verification and validation.
S5 Post market activities and governance.

Since the MDR does not replicate the structure of a product development cycle directly, the analysis needed to match the components of the MDR to the particular development steps in a suitable way. Basically, the MDR is structured into Recitals and Articles as well as Annexes. Considering a product development approach, the Recitals refer to basic requirements that need to be implemented within the regulatory system. Thus, they are related to the definition of basic goals (S1) as well as the requirements engineering and specification steps (S2). The Articles and Annexes provide the detailed information on how the system is implemented. Regarding the regulatory system, these components are not specifications but already parts of the implementation of the regulatory system (S3). Additionally, this implementation is accompanied by further elements, e.g., additional documents, tools and infrastructure like guidances, (harmonized) standards, databases, authorities, and other relevant organizations. These components were included in the analysis, where necessary.

**Figure 1:**
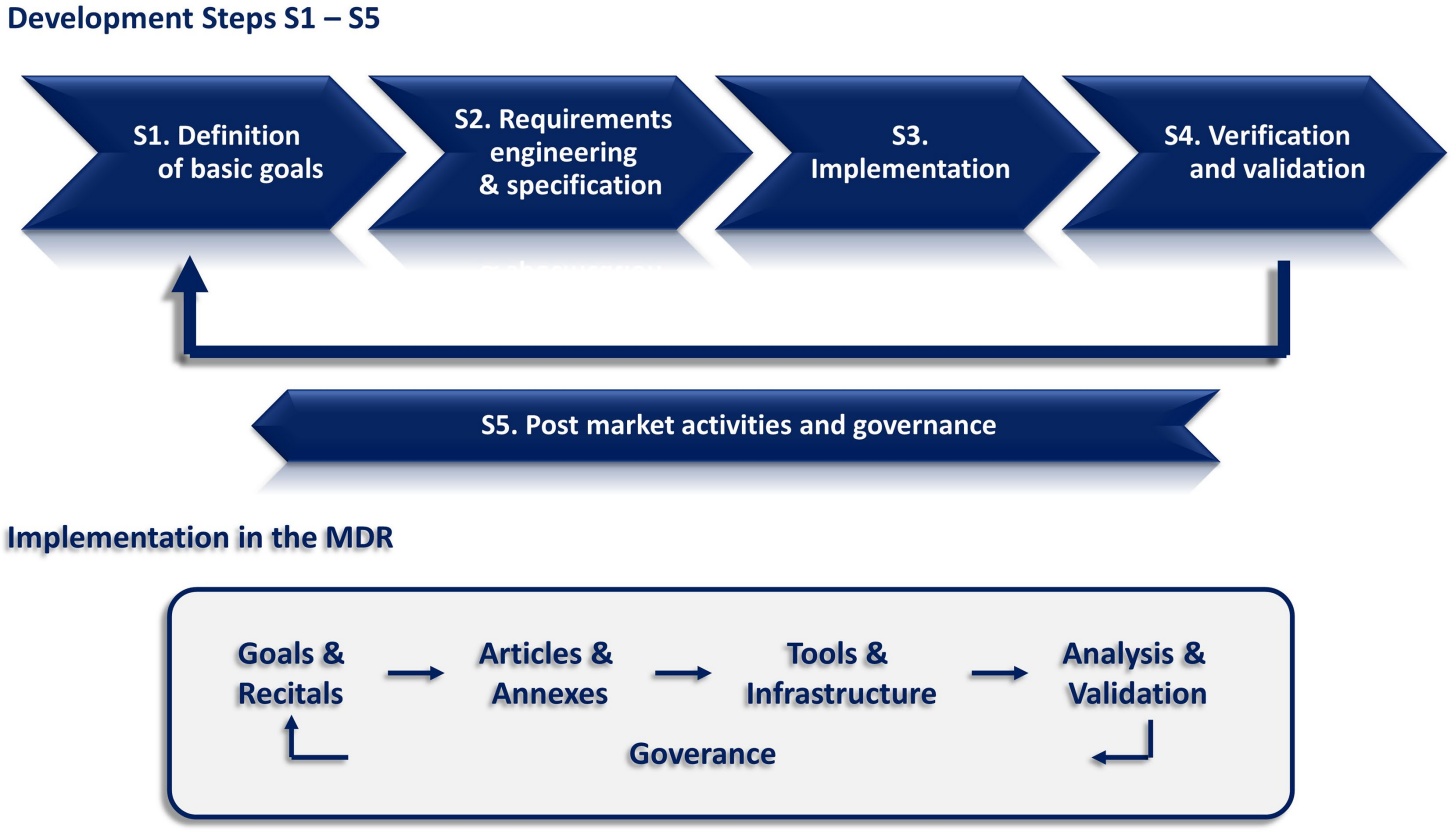
Upper row: development steps (S1–S5) considered for the analysis regarding a consequent implementation of the MDR. Bottom row: elements in the MDR and its regulatory system for implementing these development steps.

Following this approach, our analysis was based on the following correspondences between the product development steps and the components of the MDR and its regulatory system. Additionally, the development steps and their relationship to particular elements in the MDR and its regulatory system are visualized in Figure 1.

S1 Definition of basic goals:

Information about goals provided in the Recitals of the MDR.

S2 Requirements engineering and specification:

Recitals of the MDR and partially also Articles of the MDR.

S3 Implementation:

Concrete regulatory requirements defining the regulatory system as given in the Articles and Annexes as well as additional documents, tools, and infrastructure provided to implement the requirements in the MDR.

S4 Verification and validation:

Secondary information on how well the requirements of the MDR were implemented and the goals achieved.

S5 Post market activities and governance:

Concrete regulatory requirements defining post-market/governance activities for the regulatory system itself. This includes the corresponding Articles and Annexes as well as additional documents, tools, and infrastructure provided to implement these requirements.

**Figure 2:**
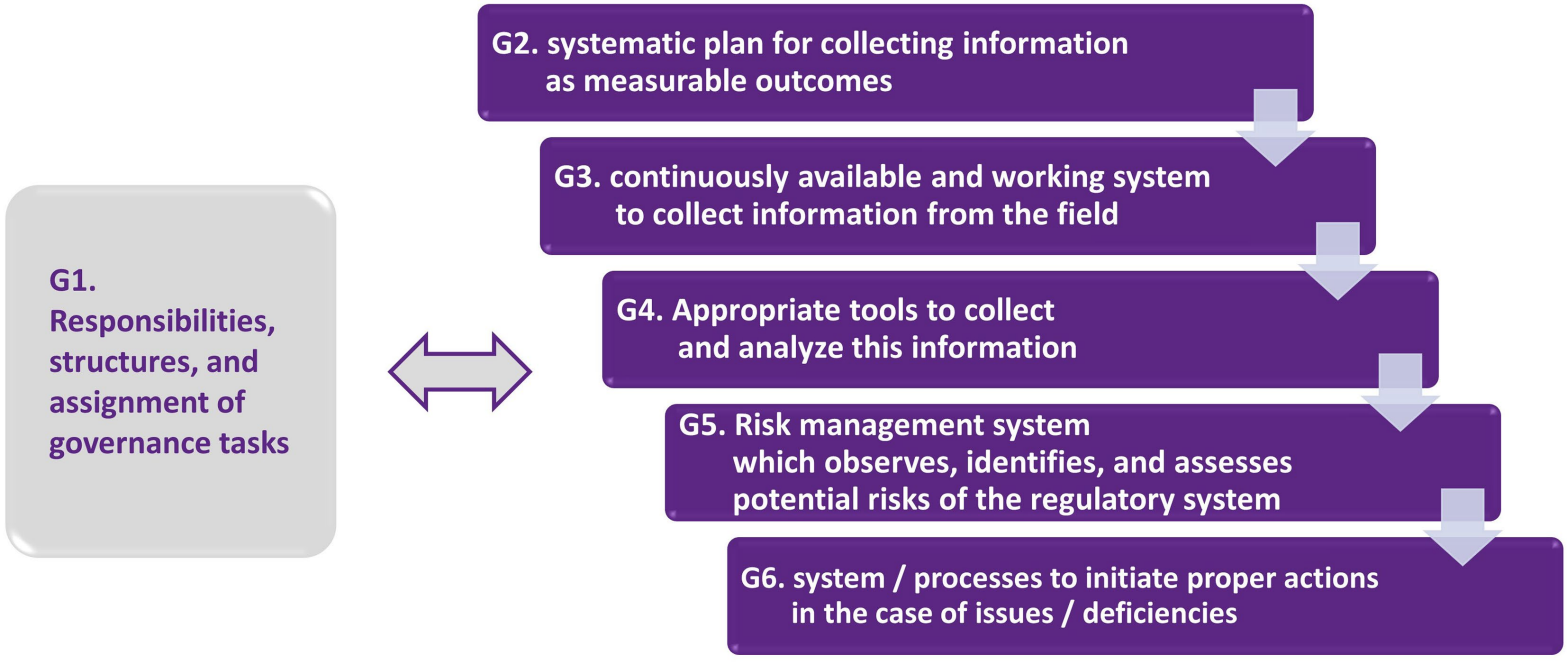
Subtasks in the governance system that should be implemented in a regulatory system.

Since governance is an important part on the organizational level, we included important steps for this in our analysis. Regarding a proper governance system, the following subtasks should be implemented in a regulatory system. These subtasks are also visualized in Figure 2. They are aligned with the requirements that the MDR and corresponding harmonized standards like the ISO 13485 demand for quality systems. In the MDR, this is focused on the quality systems to be implemented by the manufacturers of medical devices. Following the basic approach of our study, we transfer these requirements to the governance of the regulatory system itself.

G1 Definition of responsibilities, implementation of appropriate structures, and assignment of concrete tasks regarding the implementation of governance activities.
G2 A systematic plan to collect information as measurable outcomes that are able to assess the basic objectives of the regulatory system.
G3 A continuously available and working system to collect information from the field regarding potential issues and options for improvement.
G4 Appropriate tools (and/or other measures) to collect and analyze this information.
G5 An approach/a risk management system that observes, identifies, and assesses potential risks, i.e., the impact of the regulatory system on all relevant areas (e.g., the healthcare system, the economy, and society in general).
G6 A system to initiate proper actions in case of systematic issues, substantial changes in the environment of the regulatory system (e.g., in technological, clinical, economic, or organizational aspects), and options for improvement (including preventive but also corrective action). This requires appropriate elements to adjust the regulatory system in a reasonable amount of time. The system needs to be flexible enough to allow these adaptations but also robust enough that it remains reliable enough for the involved actors.

**Figure 3:**
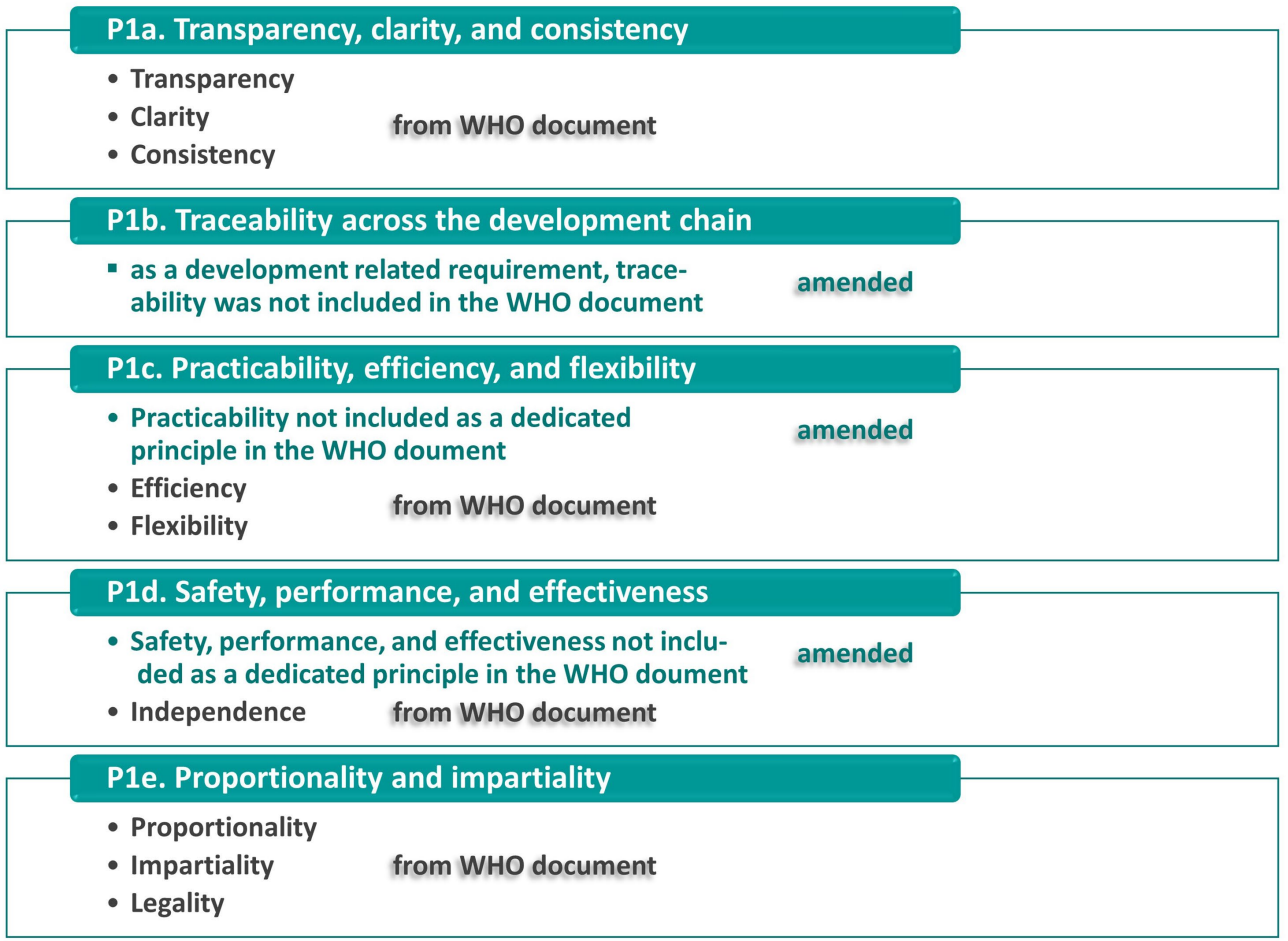
Relationship between the principles for regulatory systems in the field of medical products according to World Health Organization (18) and the principles (P1.a) to (P1.e) used in this paper. The turquoise fields represent the principles in this paper, whereas the black items in the boxes demonstrate the relationship to the principles of the WHO document (18). Amendments to the WHO approach are given in the turquoise text elements.

Further on, the analysis had to consider fundamental principles that are usually required for product development as well as regulatory systems in the field of medical devices. In particular, this refers to the following elements. The list was based on the WHO document (18) about good regulatory practices regarding the regulation of medical products. In comparison to World Health Organization (18), this paper used a different composition of these principles. However, the central principles are covered in our list. The relationship between the principles in the WHO document (18) and the principles (P1.a) to (P1.e) used in this paper is represented in Figure 3.

P1 Basic principles for the setup and implementation of products/the regulatory system.

a) Transparency, clarity, and consistency of the regulatory systems including their basic objectives, terms, and requirements.

i.e., the Recitals, Articles, and other defining parts of the MDR need to provide information in an accessible, clear, and consistent way.

b) Traceability of requirements across the development chain:

This means that the goals and requirements need to be clearly stated in the Recitals of the MDR and they are subsequently implemented in a consequent manner. Basically, this refers to the Articles and Annexes as well as further documents, tools, and infrastructure (where required). Additionally, the implementation needs to be verified and validated. The traceability needs to be maintained across the lifecycle of the MDR.

c) Practicability, efficiency, and flexibility of the regulatory system:

The regulatory system should be implemented in a way that enables all stakeholders and participants to use the system in a practical and efficient way. This includes that appropriate tools and infrastructure are provided. They shall allow to efficiently put the requirements into practice, i.e., to enable an easy application to specific use cases. For this purpose, flexibility of the system needs to be incorporated to address different medical devices and corresponding scenarios, appropriately.

d) Safety, performance, and effectiveness of the regulatory system:

The regulatory system needs to achieve its goals in a consequent manner. This includes safety, performance, and effectiveness of the regulatory system itself. This means that a positive impact on the healthcare system and its relevant actors, e.g., manufacturers, authorities, healthcare organizations, patients, etc., needs to be guaranteed. This includes a positive relationship between the benefits, e.g., achieved level of product safety or reliability for economic actors, and potential risks of the regulatory system itself, e.g., lack of availability of devices or economic impact. This item also includes legality, i.e., a sound legal basis and a reliable framework for the involved actors.

e) Proportionality and impartiality:

The requirements in a regulatory system should be proportionate, i.e., the different objectives of the regulation should be brought into balance. This is needed since particular objectives often have an antagonistic impact. For example, this applies to the objectives of product safety and availability of medical devices to ensure an appropriate treatment of diseases. This includes the proportionality regarding risks and benefits as well as the regulator’s capacity to implement and enforce measures. Additionally, impartiality refers to the equitable and fair treatment of all involved parties.

P2 Design transfer principles:

We include this category to emphasize that not only the text of the MDR needs to be considered, but also the further transfer into the operating phase of the regulatory system as a whole. This refers to the question whether appropriate tools and infrastructure are provided that ensure a high level of performance as well as practicality of the system. For medical devices, central elements of the operating phase, e.g., production processes and central elements of the post-market monitoring, need to be developed and provided with the product itself. The same should apply to the regulatory system itself. This includes the following aspects.

a) Consequent transfer of the requirements into tools and infrastructure, e.g., including required databases, (harmonized) standards, authorities, and other organizations for the implementation of the regulatory system.
b) Provision of processes regarding the development cycle and governance of the system, including risk management, quality management, complaint handling, corrective and preventive action.

**Figure 4:**
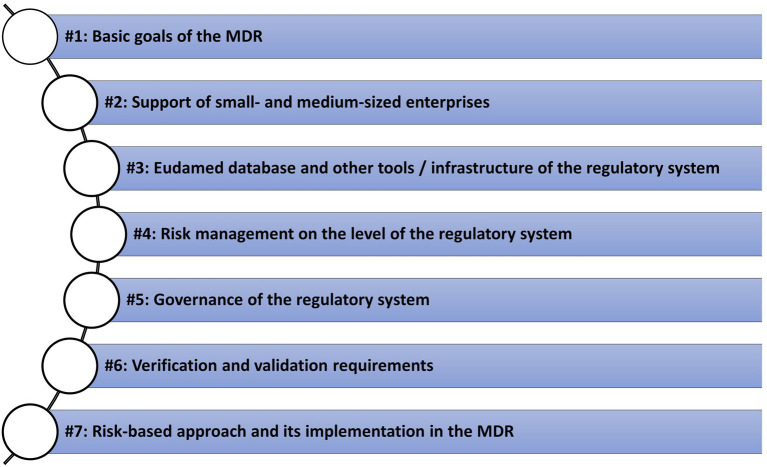
Central examples/topics for the analysis of the MDR and its regulatory system utilized in this paper.

Based on this rationale, the MDR and its accompanying elements were analyzed. It was evaluated how well these central requirements for product development were met when they were applied to the MDR. In particular, the aspects shown in Figure 4 were analyzed as central examples.

## 3 Results

Within the analysis, a series of deficiencies were found. This refers to deviations between the MDR and basic requirements from standard product development policies, as they are also posed on medical devices by the MDR itself. According to the exemplary character of the analysis, we present central aspects that demonstrate such deviations. We demonstrate the results using the seven areas presented in Figure 4

### 3.1 Example 1: basic goals of the MDR

As a first topic, we analyzed the basic goals of the MDR and the question how they are anchored in this regulation. The definition of the goals is related to step S1 according to our nomenclature. But, also the other steps (S2–S5) need to be considered regarding the achievement of these goals.

As already mentioned, the definition of the goals had to be extracted from the Recitals. In Recital 2, the MDR includes the following statement as its main objective: “This Regulation aims to ensure the smooth functioning of the internal market as regards medical devices, taking as a base a high level of protection of health for patients and users, and taking into account the small- and medium-sized enterprises that are active in this sector. At the same time, this Regulation sets high standards of quality and safety for medical devices in order to meet common safety concerns as regards such products. Both objectives are being pursued simultaneously and are inseparably linked whilst one not being secondary to the other.“.

This substantiates that the MDR has an overarching goal and that smooth functioning as well as quality and safety are the main objectives. These aspects are related to the principles of practicability (P1.c) and safety (P1.d). According to Recital 2, both aspects are mainly related to the medical device sector. The overall impact on the healthcare system also in terms of positive effects, e.g., the availability of medical devices to enable adequate treatment of diseases, is not explicitly addressed. The safety aspects are mainly related to product safety and not regarding safety and performance of the healthcare system in general.

The definition of the goals in the MDR remains on this broad and imprecise level. The goals are not transparently linked to more detailed specifications. Additionally, no concrete performance indicators or other parameters for a systematic evaluation are provided in the MDR (S2). This impedes a consequent analysis whether these goals have been actually achieved with respect to the implementation in the Articles or other components (S3). This also makes a thorough verification and validation (S4) almost impossible. Such steps are not provided in the MDR, and to the best of our knowledge no accompanying documents are available that include an appropriate analysis. An evaluation of the outcomes including the achievement of the MDR’s goal is defined in Article 121. But it is only addressed for May 27, 2027, i.e., 10 years after the publication of the MDR. As already mentioned, this was shifted slightly forward to the targeted evaluation in 2025 (13). Such an evaluation is related to steps S4 and S5. For medical devices, it has to be delivered before the product is placed on the market. Based on requirements for post market surveillance, the re-evaluation needs to take place in a regular time frame, usually every 1–2 years, depending on the risk class of the device.

Thus, the MDR lacks several central requirements it poses on medical devices. Besides the missing/late evaluation, it also lacks clarity and precise criteria, violating principle (P1.a). This makes it almost impossible to rate the success of the MDR. The weak measures to implement a systematic evaluation and corresponding governance of the system are related to deficiencies with respect to principles (P2a) and (P2b). Additionally, no traceability (P1.b) between central goals and further development steps (e.g., further Recitals, Articles, or other components) is provided. This impedes a consequent check across the chain of development steps.

### 3.2 Example 2: support of small- and medium-sized enterprises

As already mentioned, Recital 2 defines the “smooth functioning of the internal market as regards medical devices” as a central requirement, “taking into account the small- and medium-sized enterprises that are active in this sector.” In our analysis, we checked the consistent implementation of this goal. As a first step, this raises the question of whether the consideration of small- and medium-sized enterprises (SMEs) is actually transferred into adequate specifications. Basically, there are four places where the term “small- and medium-sized enterprise” or also “micro and small enterprises” is explicitly used in the further course of the MDR.

- Recital 95, stating that “a sufficient transitional period for that adaptation and for the organizational arrangements that are to be made” to allow (inter alia) economic operators, especially SMEs “to adapt to the changes introduced by this Regulation and to ensure its proper application.”
- Article 15 stating that SMEs “shall not be required to have the person responsible for regulatory compliance within their organization but shall have such person permanently and continuously at their disposal.”
- Article 106 stating that the fees payable to the Commission for the advice provided by expert panels and expert laboratories shall be reduced when an SME is involved as a manufacturer.
- Annex VII, section 1.2.8 stating that notified bodies “shall operate in accordance with a set of consistent, fair and reasonable terms and conditions, taking into account the interests of small and medium-sized enterprises.”

This leads to the following observations.

- There is no clear link between the basic objective defined in Recital 2 and the further paragraphs where SMEs are mentioned. Additionally, the listed places do not provide sufficient evidence that the needs of SME are sufficiently covered and that a smooth functioning is appropriately addressed, especially for this group. This violates the principles clarity (P1.a), traceability (P1.b), and practicability (P1.c).
- Articles 15 and 106 as well as Annex VII, section 1.2.8 propose particular benefits for SMEs. However, other aspects that may be considered important are not further specified. Based on this, it cannot be judged whether these specific measures sufficiently address the needs of SMEs. Additionally, Article 106 does only apply in special cases where an expert panel or expert laboratory is involved. Thus, an actual validation (S4) regarding the fulfillment is not possible.
- Regarding Recital 95, an implementation is provided in Articles 120 and 123 defining the entry into force of the MDR and its transitional periods. This addresses steps (S2) and (S3). But it remains open, whether the implementation achieved a valid goal (S4), i.e., whether a reasonable transitional period was achieved. Several preparation steps (e.g., availability of notified bodies and other infrastructure like harmonized standards) appeared to have no sufficient transitional period. At least, this applies to the initial release of the MDR in May 2017. This is documented by the prolongation of the translation periods that were implemented later on for the MDR. Based on this, we have to state that the initial requirements did not represent an acceptable/valid goal, violating the requirement for a validation step (S4) as well as principle (P1.c). Additionally, the translation periods do not only affect SMEs but manufacturers, in general. Thus, they do not represent specific measures for SMEs.
- It should be mentioned, that the adjustments were implemented after pressure from the market. Thus, some governance actions, referring to step (S5) and principle (P2b), were pursued. However, no clear escalation procedures are defined in the MDR that systematically enable appropriate and timely reaction to such issues (see also further analysis steps in section 3.5).

In summary, the goal to support SMEs defined in Recital 2 lacks clarity and traceability, violating principle (P1.a). Some other Recitals and Articles provide particular implementation steps. But, they again remain vague and it remains open whether this sufficiently covers the actual needs of SMEs. Thus, the fulfillment of principle (P1.c) regarding practicability and efficiency cannot be sufficiently rated. Additionally, the MDR does not include any information about the proportionality and impartiality of the implemented steps with regard to principle (P1.e). This refers to the question of whether the specific measures for SMEs achieve a balance between required effort and admissible benefits for SMEs. Further on, it remains unclear which amount of benefits for SMEs can be considered fair in relation to other actors. At the end, the MDR does not provide evidence that the goal was met in an appropriate way.

### 3.3 Example 3: Eudamed database and other tools/infrastructure of the regulatory system

Several Recitals and Articles of the MDR are related to a European database on medical devices (Eudamed), e.g., Recitals 41–47 and 67, Articles 25–33, and also some of the Annexes. In this case, we did not analyze the details of the requirements and specifications. Instead, we focused on the question of how consequently the associated tools were provided. This is basically related to step (S3) and principle (P2a) regarding a proper design transfer as well as implementation and provision of required tools. For a medical device, it is required that all relevant accessories are available and working appropriately. This is crucial to ensure the proper functioning of the device including all the relevant equipment. Without fully specified, implemented, tested, and validated tools, a medical device may not be placed on the market.

For the MDR, the functionality of the Eudamed database was neither fully specified nor implemented, tested, or validated, even a substantial time after the entry into force of the MDR (5, 10, 11). Thus, the steps (S2) to (S4) were not properly addressed. As already mentioned, this would not allow to place the MDR on the market according to its own rules. In deviation to the rules for medical devices, the MDR introduces a grace period until the tool gets available. In this direction, Recital 96 states: “In order to ensure a smooth transition to the new rules for registration of devices and of certificates, the obligation to submit the relevant information to the electronic systems set up at Union level pursuant to this Regulation should, in the event that the corresponding IT systems are developed according to plan, only become fully effective from 18 months after the date of application of this Regulation.” However, a sufficiently/comprehensively functioning Eudamed database was not available, even a significant amount of time after the scheduled date (3, 5, 10, 11). Thus, there definitely was a substantial discrepancy between the requirements in the MDR and the provision of the Eudamed database as an accessory for the MDR. Again, this compromises at least the steps (S3) to (S4) as well as principle (P2a).

Subsequently, also the goal for a smooth functioning of the market (see, e.g., Recital 2) is undermined, violating the principle of practicability (P1.c). In this context, not only the MDR itself needs to be considered but the entire regulatory system built on it. Without properly working tools and infrastructure, a regulatory system cannot achieve a goal like a smooth functioning of the market.

Besides the Eudamed database, this applies to other components that are crucial for the regulatory system. According to the structure and the specifications of the MDR, this refers to elements like the availability of guidances to clarify central requirements, harmonized standards, or notified bodies. These tools and infrastructure also had a substantial delay that compromised the practicability and the smooth functioning of the market (6, 7, 10, 11, 16), even though the MDR stated, e.g., in Recital 95: “It is also particularly important that, by the date of application of this Regulation, a sufficient number of notified bodies be designated in accordance with the new requirements so as to avoid any shortage of medical devices on the market.”

In this context, it has to be mentioned that notified bodies as well as manufacturers need a sufficiently designed transition period. For the notified bodies, this includes the preparation of their own processes as well as the notification procedures. For manufacturers, this applies to the availability of information on how to design their products and how to pursue the conformity assessment. For achieving a high level of quality, the integration of regulatory requirements should be addressed early in the development phase. For products with a development time of, e.g., 12–24 months, this requires a corresponding lead time for the clarification of central regulatory requirements and also a reliable provision of tools and infrastructure in the regulatory system. For the MDR, these steps were not performed in time, regarding the requirements of the actors in the medical device sector as well as according to requirements in the MDR like the 18 months for a fully effective implementation of the Eudamed database (3, 5, 10, 11, 16).

### 3.4 Example 4: risk management on the level of the regulatory system

The MDR uses risk management as a cornerstone regarding the safety of medical devices. For example, this is addressed in Recitals 32, 33, and 74 as well as Article 10 in combination with Chapter 1 of Annex I. Applying the rules of the MDR to the MDR itself, this means that systematic risk management procedures should be implemented for the regulatory system itself, i.e., the risks of the regulatory system on the relevant actors in the healthcare system, including manufacturers of medical devices, healthcare institutions, and finally patients. For example, the availability of medical devices may be compromised when the regulatory requirements are disproportionate and do not allow to develop and produce medical devices, in an economically reasonable way. Subsequently, patients cannot be treated properly when important devices are not sufficiently accessible. Even though risks to the health of individuals are the primary focus regarding the requirements in the MDR, risks to the manufacturers are an important part. If the manufacturers cannot act economically reasonably, the healthcare system is affected in general. Thus, this has to be considered as a substantial risk with respect to the overarching goal of a medical device regulation for supporting a smooth functioning of the market but also regarding the reliability and performance of the entire healthcare system.

According to Annex I(3) of the MDR, risk management steps include the provision of a risk management plan, the identification and analysis of known and foreseeable hazards, estimation and evaluation, as well as the elimination or control of risks. Finally, the particular and the overall risks need to be reduced as “far as possible without adversely affecting the benefit–risk ratio.” For example, product safety should be considered as a key element of the regulatory system. However, it must not dominate other aspects like the availability of medical devices that are important to ensure a high-level quality of the overall healthcare system. In general, this demonstrates the interaction between the principles of practicality, efficiency, and flexibility (P1.c) on the one side, and safety, performance, and effectiveness (P1.d) on the other side. At the end, these partially antagonistic goals need to be balanced according to the principle of proportionality and flexibility (P1.e). Regarding its practical implementation, it also addresses the consequent transfer of requirements into tools and infrastructure, including the development and operational phase of the system, i.e., principles (P2a) and (P2b).

In this regard, risk management procedures are not only relevant during the development phase. According to the MDR, “risk management shall be understood as a continuous iterative process throughout the entire lifecycle of the device” – or in this case, the regulatory system. Thus, potential issues and risks of the system need to be monitored during the operational phase. This includes a systematic implementation of processes for monitoring issues and reacting to them in an appropriate way. For example, the MDR states in Recital 74: “Manufacturers should play an active role during the post-market phase by systematically and actively gathering information from post-market experience with their devices … To this end, manufacturers should establish a comprehensive post-market surveillance system, set up under their quality management system and based on a post-market surveillance plan. Relevant data and information gathered through post-market surveillance, as well as lessons learned from any implemented preventive and/or corrective actions, should be used to update any relevant part of technical documentation, such as those relating to risk assessment and clinical evaluation, and should also serve the purpose of transparency.”

The MDR itself does not include an analysis of its own risks, even though it sometimes mentions the impact on actors. It primarily focuses on product safety and thus the risk to patients and other users. It also mentions that economic actors, especially SMEs, should be taken into account. However, it does not clearly state such risks. It also does not transparently link the risks to measures, e.g., specific Articles. Additionally, there is no consequent analysis of the risks and their impact, in the MDR. To the best of our knowledge, there also are no accompanying documents that include such an analysis. In our search, we only found the documents (19–21) that include an impact assessment and a rationale about different options for implementing the MDR. However, these documents date back to Sep 2012, i.e., more than 4 years before the publication of the MDR.

Likewise, the MDR does not include systematic processes for analyzing its own risks, collecting issues, and reacting to them. Instead, identification and documentation of issues mainly was performed by actors from the field, including manufacturers/trade unions, researchers, non-governmental organizations, see, e.g., (4–7, 10, 11, 14–16). Only Article 121 of the MDR refers to a dedicated action for post-market activities. However, an evaluation 10 years after the publication of the regulation cannot appropriately address the requirements regarding impact assessment, collection of issues during the operational phase, implementation of corrective and preventive actions, or other processes that need to be available to monitor medical devices.

In summary, almost no governance processes are specified in the MDR, regarding the safety, performance, and effectiveness of the regulatory system. None of the steps (S1) to (S5) was addressed as far as this can be extracted from the MDR or accompanying information. This substantially violates the principle of safety (P1.d) regarding the impact of the regulatory system itself. Additionally, the design transfer principles (P2a) and (P2b) are not addressed systematically. This also compromises the principles of transparency (P1.a) as well as traceability (P1.b), practicability (P1.c), and proportionality (P1.e).

### 3.5 Example 5: governance of the regulatory system

In general, governance structures are important elements for products, manufacturers, and other organizations to ensure high quality as well as long-term success of their systems. To address this for medical devices, the MDR states in Recital 74: “Manufacturers should play an active role during the post-market phase by systematically and actively gathering information from post-market experience with their devices in order to update their technical documentation and cooperate with the national competent authorities in charge of vigilance and market surveillance activities. To this end, manufacturers should establish a comprehensive post-market surveillance system, set up under their quality management system and based on a post-market surveillance plan. Relevant data and information gathered through post-market surveillance, as well as lessons learned from any implemented preventive and/or corrective actions, should be used to update any relevant part of technical documentation, such as those relating to risk assessment and clinical evaluation, and should also serve the purpose of transparency.”

This extends to the quality management system in general. For example, this is constituted in Article 10(9) of the MDR: “The quality management system shall cover all parts and elements of a manufacturer’s organisation dealing with the quality of processes, procedures and devices. It shall govern the structure, responsibilities, procedures, processes and management resources required to implement the principles and actions necessary to achieve compliance with the provisions of this Regulation.” Such requirements are also included in ISO 13485 as the harmonized standard for quality management systems in the context of medical devices. According to this, the subtasks (G1) to (G6) should be implemented by the MDR and its regulatory system as presented in Chapter 2.

**Figure 5:**
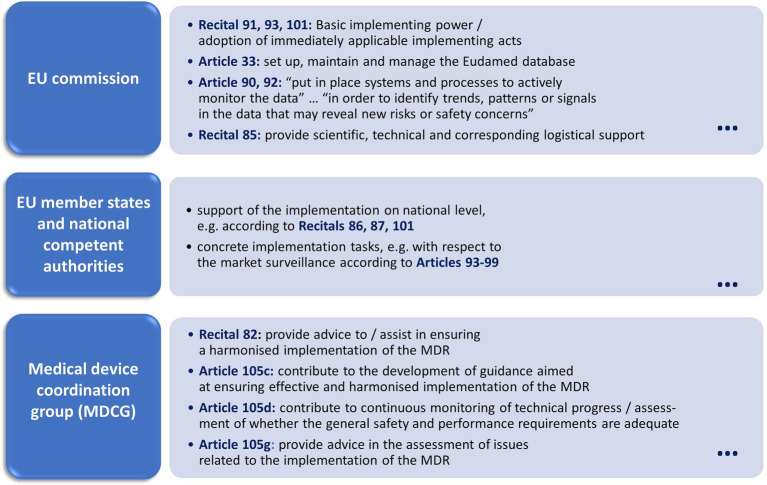
Overview of main actors regarding their responsibilities for governance of the MDR and its regulatory system.

Regarding the definition of responsibilities, i.e., subtask G1, the MDR includes different actors in the regulatory system. It also assigns particular tasks to these actors. This includes the following actors and assignments. Figure 5 provides a visual overview about this.

- *EU commission*, e.g.,

a) Basic implementing power for the MDR and adoption of immediately applicable implementing acts (Recital 91, 93, and 101).
b) “Set up, maintain and manage the European database on medical devices (‘Eudamed’)” (Article 33) as well as “put in place systems and processes to actively monitor the data available in the electronic system referred to in Article 92, in order to identify trends, patterns or signals in the data that may reveal new risks or safety concerns” (Article 90).
c) “Provide scientific, technical and corresponding logistical support to coordinating national authorities and ensure that the regulatory system for devices is effectively and uniformly implemented at Union level based on sound scientific evidence” (Recital 85).

- *EU member states and national competent authorities*.

a) Support of the implementation on national level, e.g., Recitals 86, 87, 101 and further Recitals regarding concrete implementation tasks as well as corresponding Articles, e.g., Articles 93–99 regarding the market surveillance of medical devices.

- *Medical Device Coordination Group (MDCG)*, basically to “provide advice to the Commission and to assist the Commission and the Member States in ensuring a harmonised implementation of this Regulation” (Recital 82). According to Article 105, this includes the following selected subtasks.

c) “To contribute to the development of guidance aimed at ensuring effective and harmonised implementation of this Regulation, in particular regarding the designation and monitoring of notified bodies, application of the general safety and performance requirements and conduct of clinical evaluations and investigations by manufacturers, assessment by notified bodies and vigilance activities;”
d) “To contribute to the continuous monitoring of technical progress and assessment of whether the general safety and performance requirements laid down in this Regulation and Regulation (EU) 2017/746 are adequate to ensure safety and performance of devices, and thereby contribute to identifying whether there is a need to amend Annex I to this Regulation;”
g) “To provide advice, either on its own initiative or at request of the Commission, in the assessment of any issue related to the implementation of this Regulation.”

Based on this, different actors shall contribute to the governance of the regulatory system. However, these tasks remain vague and subsequently important steps remain deficient. As a first step, this applies to the setup of a concrete plan and relevant criteria for guiding the system, as given in subtask (G2). The MDR does not provide a clear plan and corresponding criteria, e.g., regarding monitoring and evaluation of the system. To the best of our knowledge, no corresponding documents are available from the relevant actors, in particular from the MDCG as the institution with central responsibilities according to Article 105. At most, appropriate analysis steps are provided by trade unions, researchers, or other organizations (4–7, 10, 11, 14–16), but not by the responsible institutions. Without the definition of a systematic plan and corresponding criteria, the subsequent steps remain deficient. This applies to a systematic collection of data (G3) and a system to implement this and evaluate the data (G4). It also refers to the analysis of potential issues and their risks (G5) as well as potential corrective and preventive actions (G6).

Without these steps and without a regular analysis of its performance, governance of the regulatory system cannot be achieved. In particular, this violates the principles of safety, performance, and effectiveness (P1.d) as well as the design transfer tasks (P2a) and (P2b). Based on this, issues in the regulatory are neither systematically monitored nor properly escalated and addressed. The MDR also shows deficiencies with respect to principles like transparency (P1.a) as well as steps (S4) and (S5). For this purpose, it is not sufficient that 10 years after starting the operational phase of the MDR, an evaluation is addressed, according to Article 121 of the MDR. The shift of the targeted evaluation is to 2025 does not resolve this deficiency (13). Additionally, it is not sufficient that some aspects, like the post-marketing monitoring of issues with concrete devices, have more detailed requirements, e.g., according to Articles 93–100. Additionally, these post-market requirements basically refer to the monitoring of devices and not to the governance of the regulatory system itself. Based on these findings, it has to be stated that specification of governance steps (S2) is limited and implementation (S3), verification and validation (S4), as well as post-market surveillance/governance of these activities (S5) are almost not accessible, at least in terms of activities that were made transparent by the responsible actors.

### 3.6 Example 6: verification and validation requirements

In the MDR, verification and validation activities, i.e., step (S4), are constituted as an important element to guarantee the success of a product. In Annex II – Technical Documentation, the MDR states: “The documentation shall contain the results and critical analyses of all verifications and validation tests and/or studies undertaken to demonstrate conformity of the device with the requirements of this Regulation and in particular the applicable general safety and performance requirements.” If we apply this requirement to the MDR itself, this means that it needs to be demonstrated that the MDR actually achieves its objectives and requirements. Additionally, this requires transparency and clarity of requirements as well as their traceability, i.e., principles (P1.a) and (P1.b). Traceability needs to encompass the entire development chain, i.e., steps (S1) and (S4), and its continuation to the operational phase, i.e., step (S5). The need for appropriate verification and validation activities is also required by ISO 13485 (22) as the harmonized standard regarding quality management systems for medical devices. In general, these activities need to be performed and documented before the product is placed on the market.

In the context of medical devices, this need does not only include the device itself but also all tools, processes, and other infrastructure components that contribute to the success of the device. This is related to the design transfer principles (P2a) and (P2b). For an organization or a system in general, deficiencies regarding the validity of a system may be highly detrimental regarding the functioning as well as the safety and performance of the system, i.e., regarding principles (P1.c) and (P1.d). For example, a change in basic processes in a company, a production system, an IT system, etc. usually has a high impact on the entire organization. Thus, new processes and also updates of processes need to be validated systematically. Additionally, this has to be provided before the product is placed on the market, according to the MDR and ISO 13485. ISO 13485 allows a risk-based approach for this. This means that the effort regarding these activities can be adapted to the risk profile of the particular process or tool.

Since the impact of a regulation in the field of medical devices on the affected healthcare systems as well as on the medical device industry as an important economic sector has to be rated high, the verification and validation activities should be pursued in a systematic and consequent way. For example, this would include the already discussed examples, containing the following topics.

- How well is the smooth functioning of the internal market with respect to medical devices actually supported by the MDR and its regulatory system?
- How far is it guaranteed that the interests of small- and medium-sized enterprises are appropriately addressed?
- How far does Eudamed fulfill the requirement from Recital 46: “Eudamed’s electronic systems regarding devices on the market, the relevant economic operators and certificates should enable the public to be adequately informed about devices on the Union market”?
- How far was/is the following requirement from Recital 95 fulfilled and proven: “To allow economic operators, especially SMEs, notified bodies, Member States and the Commission to adapt to the changes introduced by this Regulation and to ensure its proper application, it is appropriate to provide for a sufficient transitional period for that adaptation and for the organisational arrangements that are to be made.”
- How positive or negative does the strict requirement for the provision of clinical data (MDR Article 61) influence the market, including the safety but also the availability of products?
- How far was/is the following requirement from Recital 85 fulfilled and proven: “The Commission should provide scientific, technical and corresponding logistical support to coordinating national authorities and ensure that the regulatory system for devices is effectively and uniformly implemented at Union level based on sound scientific evidence.”

To the best of our knowledge and our efforts to search for appropriate information, the responsible actors have not performed such activities. At least, they are not systematically documented. According to the principle “not documented, not done,” we have to assume that they have not been performed at all. Based on this, it has to be stated that most of the decisions in the legislative process were made without clear evidence. According to the requirements in the MDR itself, this lack of verification and validation activities appears to be inadequate in the field of medical devices – in particular, regarding the high impact the MDR has. If such evidence cannot be provided comprehensively at the start of the operational phase, at least appropriate measures would need to be implemented to approach this in the best way possible, e.g., by implementing appropriate post-market activities. However, an evaluation 10 years after placing the system on the market, according to Article 121, or 8 years, according to the targeted evaluation performed in 2025 (13), cannot be considered sufficient.

In summary, this again violates basic principles of product development as well as the provision of regulations in the field of medical devices. In particular, this applies to the principles of:

- Transparency, clarity, and consistency (P1.a), e.g., regarding deficiencies in the comprehensive provision of terms and requirements. Finally, this compromises the possibility to perform concise verification and validation activities since these activities rely on clearly elaborated criteria.
- Traceability (P1.b) – compromising a consequent implementation and tracking of verification and validation activities.
- Practicability, efficiency, and flexibility (P1.c) – since the practical success and smooth functioning of the regulatory system cannot be rated and guaranteed without proper testing, analysis, and implementation of required improvements.
- Safety, performance, and effectiveness (P1.d) – since again clear criteria are missing that are important to rate the impact of the system and its associated risks.

As already mentioned, all steps are included in this regard, including the entire development chain, i.e., steps (S1) and (S4), as well as the continuation to the operational phase step, i.e., step (S5).

### 3.7 Example 7: risk-based approach and its implementation in the MDR

At several places, the MDR states that central activities should take the potential risks of medical devices into account, e.g., regarding the risk classification and the subsequent application of regulatory requirements. For example, this refers to the proportionality between the risks or the risk class of the device on the one side and conformity assessment procedures, the provision of clinical evidence, or post market activities on the other side. In the next sections, we analyze how consequent this approach is pursued in the MDR. As a first step, we address the implementation of the risk classes and their relationship to the risks of the actual device in section 3.7.1. As a second step, we analyze whether the regulatory requirements are adapted to the risks of the device in a proportionate way. This step is presented in section 3.7.2. In both cases, we use the term “risk-based approach” as a principle to pursue a proper relationship between the risks of the device and the applicable regulatory requirements. In the second step, this also includes the proportionality between the regulatory requirements and the risks that the regulatory system itself poses onto its environment and in particular, the healthcare system.

**Figure 6:**
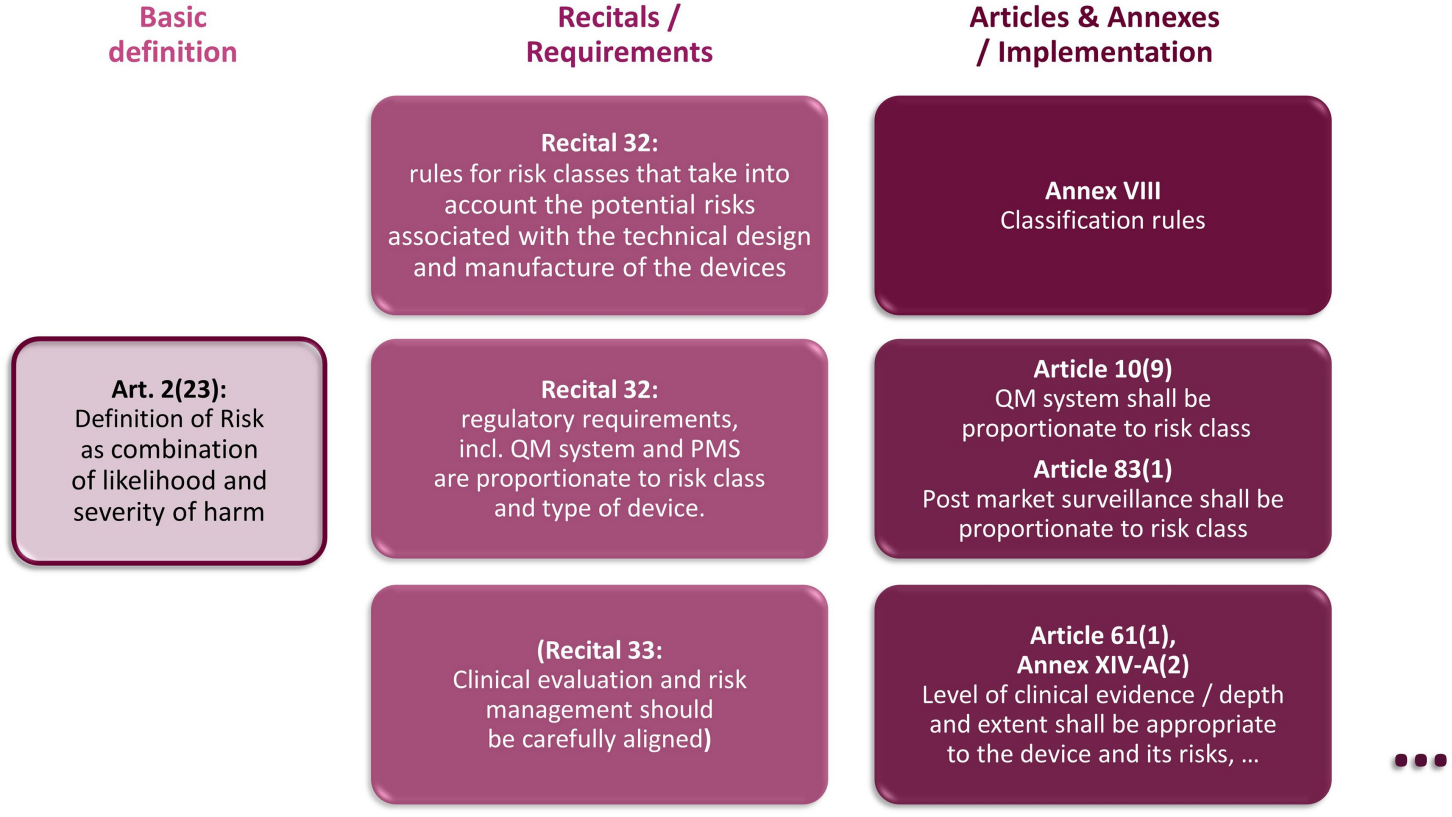
Relationship between central recitals, articles, and Annexes regarding the implementation of a risk-based approach in the MDR.

The analysis for both steps is based on the following Recitals and Articles of the MDR. An overview about the relationship between these elements is given in Figure 6.

- Definition of “risk” in Article 2(23): “‘risk’ means the combination of the probability of occurrence of harm and the severity of that harm.”
- Recital 58: “It is necessary, in particular for the purpose of the conformity assessment procedures, to maintain the division of devices into four product classes in line with international practice. The classification rules, which are based on the vulnerability of the human body, should take into account the potential risks associated with the technical design and manufacture of the devices. …”
- Recital 32: “To ensure that devices manufactured in series production continue to be in conformity with the requirements of this Regulation and that experience from the use of the devices they manufacture is taken into account for the production process, all manufacturers should have a quality management system and a post-market surveillance system in place which should be proportionate to the risk class and the type of the device in question. …”
- Article 61(1): “… The manufacturer shall specify and justify the level of clinical evidence necessary to demonstrate conformity with the relevant general safety and performance requirements. That level of clinical evidence shall be appropriate in view of the characteristics of the device and its intended purpose.”
- Annex XIV-A(2): “The clinical evaluation shall be thorough and objective, and take into account both favorable and unfavorable data. Its depth and extent shall be proportionate and appropriate to the nature, classification, intended purpose and risks of the device in question, as well as to the manufacturer’s claims in respect of the device.”

As a first result, we have to state that the MDR uses the term “risk-based” three times. But, the MDR does never actually define it. At some places, e.g., in the citations above, alternative wordings like “proportionate to the risk/risk class of the device” are used that also are considered as a risk-based approach. However, the wordings in these references substantially deviate, e.g., between Recital 58, Article 61(1), and Annex XIV-A(2). This demonstrates that terms are not introduced consequently and the formulations are also not consistent violating principle (P1.a).

#### 3.7.1 Example 7a: risk-based approach and classification rules

In the first step, we analyzed the relationship between the classification rules and the risks of the device. In this regard, the principle of proportionality and impartiality (P1.e) is a major component. In particular, the analysis refers to the question whether the assignment to risk classes and later on the applicable regulatory requirements are proportionate to the actual device, e.g., as stated in Recital 32, Article 61(1), and Annex XIV-A(2). The analysis includes the question of how consequent the development steps (S2) to (S4) were addressed, including the consistent transfer from the requirements in Recital 32 to the actual implementation of risk classification as well as the validity of the final outcome.

In order to implement a risk based-approach, Recital 58 requests the provision of appropriate risk classes that “take into account the potential risks associated with the technical design and manufacture of the devices.” Basically, the implementation of this requirement is pursued in the classification rules in Annex VIII. This does not only apply to the basic risk classes I, IIa, IIb, and III. Furthermore, some additional categories are included, e.g., sterile, reusable, or active devices. This relates to the term “types of the device,” which, e.g., is used in Recital 32 and subsequently in Articles 10(9) and 83(1).

As a first step, we analyzed whether the classification rules provided in Annex VIII actually implement the given requirements from Recital 58. This refers to the question of whether the classification rules “are based on the vulnerability of the human body” and “take into account the potential risks associated with the technical design and manufacture of the devices.” In the MDR, the classification rules basically rely on the duration of use (i.e., transient, short term, long term), the type of the device (e.g., sterile, reusable, active, invasive devices or implants), the application area (e.g., intended for contact with the central nervous system or with the heart), specific functions (e.g., intended to administer medicinal products, intended to supply energy in the form of ionizing radiation), and level of potential harm (e.g., serious deterioration of a person’s state of health, death or an irreversible deterioration of a person’s state of health) – amongst some other aspects. Particular aspects may refer to the term “based on the vulnerability of the human body” as given in Recital 58. This may be considered as a criterion representing the severity of a potential harm. Regarding the additional requirement in Recital 58 “the classification rules … should take into account the potential risks associated with the technical design and manufacture of the devices,” the situation remains unclear. Most classification rules take into account what the device is intended for, but not “the technical design and manufacture of the device.” Thus, the implementation, i.e., the classification rules, does not consequently match the requirements as given in Recital 58.

Additionally, the classification rules do not relate to the risk of the actual device. On the one hand, many of the characteristics refer to certain groups of devices and do not consider the risk of the particular device. On the other hand, some of the remaining characteristics relate to potential harms. They do not actually relate to risks since the probabilities of occurrence of these harms are not included. Instead, the definition of risk given in Article 2(23) constitutes that these probabilities are a central component of a risk.

For example, this can be demonstrated by analyzing rule 11 (software). According to this rule, almost every type of software for a medical application has to be rated at least as class IIa. However, the actual risk of particular software devices may not be that high. For example, this applies to software-based clinical decision support system that assists in the diagnosis of diseases with a low or moderate risk level. In this case, the overall level of risk may remain in a low range for this actual device. Even more, the probabilities for substantial harms may be very limited. According to the technical design of the device, the risks can be further reduced to a low level, but this does not have an impact on the risk classification at all. In summary, the overall risk of the device should be considered as low in this case. Thus, a classification as class IIa seems not to be appropriate. However, according to the generic categorization of software, it has to be classified into a higher category and subsequently fulfill substantially higher requirements as it would be needed according to the actual risk profile.

As another example, a blunt surgical instrument that is intended for contact with the central nervous system or heart (usually) has to be classified as class III according to the classification rules in Annex VIII. Subsequently, such instruments have to fulfill the same regulatory requirements as other actually high-risk class III devices, even if these devices have limited risks. Obviously, this does not really constitute proportionality to the risks of the actual device. It would be necessary that the requirements can be better adapted to the actual risk profile of the particular device and not generically to an application domain.

This demonstrates that the classification rules do not consequently implement the requirement given in Recital 58. At best, the characteristics used in the classification rules can serve as a proxy indicating a risk. But, the characteristics do not really represent a clear relationship to risks, according to the missing probability and the missing relation to the actual device. Thus, the MDR does not pursue a consequent risk-based approach regarding the classification rules. In particular, specifications like “proportionate to the risks of the device” do not have a valid implementation in the MDR. Besides the missing consistency (P1.a) and traceability (P1.b), the principle of proportionality (P1.e) is violated.

#### 3.7.2 Example 7b: risk-based approach and proportionality regarding regulatory requirements

In the second step, we focus on the proportionality of regulatory requirements and their correspondence with a risk-based approach. On a first level, this refers to the relationship of regulatory requirements and risks of a particular device. On a second level, this is addressed regarding the impact on the regulatory system in general. Such a perspective is required to rate the proportionality of requirements on a broader level.

On the first level, we analyze whether a risk-based approach is pursued in the MDR regarding the question of how well the regulatory requirements are adapted to the risk of a particular device. For example, this refers to the implementation of the quality management system and post market surveillance as requested in Recital 32 and further specified in Articles 10(9) and 83(1). Additionally, this applies to the clinical evaluation as addressed in Article 61(1) and Annex XIV-A(2). It should be mentioned that there are other Articles in the MDR that also modify regulatory requirements according to risk classes or types of devices. In particular, this refers to specific requirements for certain classes of devices like sterile, implantable, or active devices.

On the second level, we analyze the question of proportionality in a broader context, including the relationship between regulatory requirements and the risks regarding the overall regulatory system. This includes further aspects like uncertainty of information, medical necessity, practicability of studies, or also adaptations for minority groups. In this regard, the principle of proportionality and impartiality (P1.e) is a main focus of the analysis.

**Figure 7:**
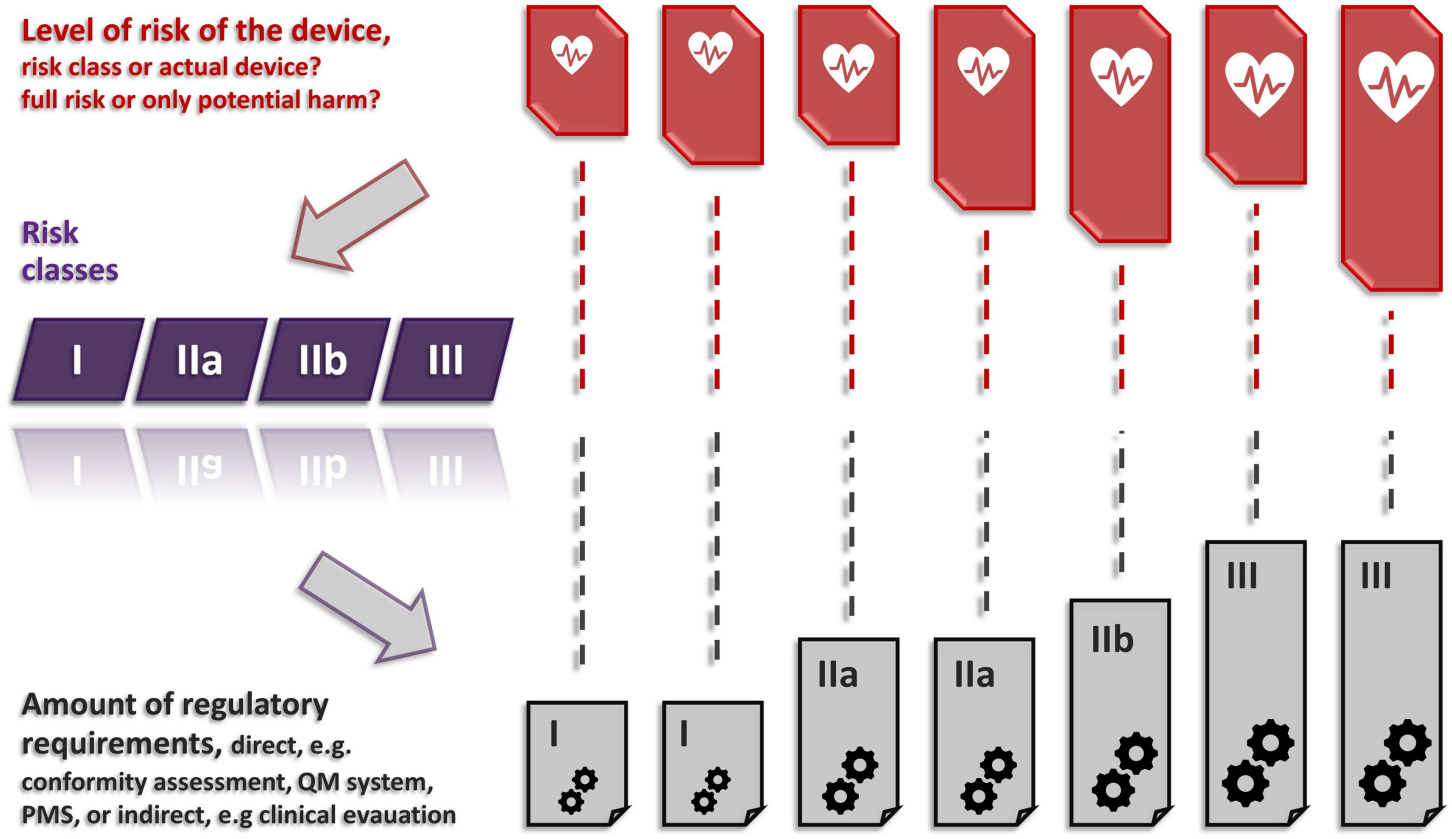
Visualization of the risk-based approach in the MDR. Instead of a direct adaptation of the level of risk to the regulatory requirements, the devices are categorized into the risk classes according to the potential harm (excluding likelihood of harms). In some aspects, e.g., conformity assessment procedures, QM system, and PMS, the regulatory requirements are then adapted to the risk classes, even if the risks of the actual device may differ.

As a first result, we have to state that Recital 32 as well as Articles 10(9) and 83(1) use the wording “proportionate to the risk class and type of the device.” Based on the findings in section 3.7.1, this translates the issue to the definition of risk classes. If the risk classes are not defined in a way that is proportionate to the risk of the actual device, the regulatory requirements addressed in Recital 32 and 10(9), 83(1), i.e., the requirements on quality management system and post market surveillance, are also not properly related to the risk of the actual device. For example, this applies to the software device or the blunt surgical instrument for the central nervous system of the heart, described in section 3.7.1. In these cases, the applicable regulatory requirements in the MDR are basically adapted to the risk class, i.e., risk class IIa or III, respectively. According to the actual risk profile of the devices, this does not seem to be proportionate to the actual risk of the particular devices. This situation is visualized in Figure 7.

Regarding the requirements for clinical evaluation, the situation differs. In contrast to the requirements on quality management system and post market surveillance, Article 61(1) and Annex XIV-A(2) require that clinical evaluation, respectively, the clinical evidence shall be proportionate to the risks of the actual device, but not to the risk class. As a side note, we have to mention that again the formulations deviate between Article 61(1) and Annex XIV-A(2). They use the wordings “shall be appropriate in view of the characteristics of the device and its intended purpose” vs. “shall be proportionate and appropriate to the nature, classification, intended purpose and risks of the device in question.” Additionally, there is no Recital that demands a proportionality between clinical evaluation or clinical evidence and the risk of the device. Only Recital 33 claims a careful alignment between risk management and clinical evaluation. Again, principles (P1.a) and (P1.b) are violated.

Especially regarding clinical evaluation, a substantial amount of complaints regarding a disproportionate burden on manufacturers has been claimed in studies and reports provided by trade unions, academia and other organizations (4–7, 10, 11, 14–16). For example, this applies to the case of surgical instruments/devices that have been on the market for a long time without major complications. Usually, it is difficult to collect clinical data for these cases. On the one hand, there are very low incentives for clinicians to perform such a study. On the other hand, also scientific journals often would not accept such a publication because of the limited scientific impact. Based on this, not enough officially recognized clinical evidence can be produced that would allow the continuation of the product according to the current MDR rules.

Subsequently, we utilize the example of these devices, i.e., instruments that have been on the market for a long time and without major complication, to analyze the principles of practicability, efficiency, and flexibility (P1.c), safety, performance, and effectiveness (P1.d) as well as proportionality and impartiality (P1.e), in these regards. It needs to be mentioned that these principles strive into opposite directions and need to be balanced against each other. For example, this applies to the relationship between practicability and efficiency on the one hand and safety on the other hand. High demands on clinical data may improve the safety, but this often reduces practicability and efficiency, e.g., as demonstrated in the example above. In order to finally judge the proportionality of regulatory requirements, a broader perspective needs to be adopted. This includes parameters like uncertainty of information about risks, medical necessity of a device, general risks to healthcare supply, or special characteristics of minority groups. This is demonstrated subsequently.

The example with the long-term established instruments, i.e., products that can already be considered as standard-of-care, demonstrates that the level of uncertainty is a major factor when rating the proportionality of regulatory requirements. The more certain we are about a given risk, the more robustness we have regarding the assessment of risks for this device. If the device is new, there usually is more uncertainty and the estimates are less reliable. If the device is on the market for a long time, the assessment gets more stable, even if there are not that many high-level clinical studies. Real-world evidence may outperform such kind of studies in certain situations. In the MDR, uncertainty regarding risks is not integrated as a relevant parameter neither in the risk classification nor in the adaptation of regulatory requirements according to a dedicated risk-based approach.

Medical necessity and/or the feasibility of performing appropriate clinical studies is another relevant parameter. In particular, this applies to vulnerable groups (e.g., children or pregnant women) and/or in the case of rare diseases. In these cases, clinical studies are often not feasible and/or economically affordable. Basically, this is due to high restrictions and burdens to include appropriate patients into these studies and/or the limited population that is available for these cases. For example, a study with pediatric patients having a remote illness is almost impossible to implement. Based on this, these groups fail to get suitable treatment – even in the case of a high medical necessity. This conflicts with the requirement for non-discrimination that is given by the EU Charter of Fundamental Rights.

Again, the regulatory requirements should be adapted to other factors than pure risks or even only potential harm (without including the associated probabilities). The relationship between risks and benefits of providing high-level clinical studies should be considered, in a more general way. This includes the risks according to the non-availability of appropriate devices and corresponding treatment options. Basically, this has to be rated based on the risk–benefit relationship within the regulatory system in general. The regulatory system needs to find a proper balance between safety as well as high-level evidence of performance, i.e., principle (P1.d), on the one side and practicability as well as flexibility, i.e., principle (P1.c), on the other side. Only then, the principle (P1.e) regarding proportionality and also impartiality, e.g., for the mentioned minority groups, can be achieved in a broad sense. Flexibility includes the possibility for alternative pathways, e.g., that use restrictions regarding the use context, stronger surveillance, or other measures to reduce potential risks on this level. For particular applications, such adaptations have been addressed in the MDCG guidance 2024-10 regarding orphan devices (23). But, they were not addressed in a more general way, in the context of the MDR.

For achieving proportionality, a systematic assessment of the impact of the regulatory requirements is crucial. Otherwise, the decisions about appropriate pathways and adequate adaptations of regulatory requirements remain deficient. Due to the lack of assessments available from the responsible actors in the regulatory system, this could not yet be addressed systematically in the MDR. Based on this, a proper balance between the principles of safety, performance, and effectiveness (P1.d) on the one side and practicability, efficiency, and flexibility (P1.c) on the other side could not be pursued in a dedicated way. At the end, this violates the principle of proportionality and impartiality (P1.e). Finally, we have to state that a risk-based approach has not been consequently implemented, in the sense that the regulatory requirements are properly adapted to the risk of the particular device as well as the risks for the environment, including the healthcare sector.

## 4 Discussion

In summary, our analysis demonstrates that the MDR contains major deficiencies when we apply its own rules to it. By a series of concrete examples, we showed that basic principles of product development, design transfer, and lifecycle management are violated. This also applies to principles for regulatory systems in the field of medical devices. Important development steps are missing or not pursued consequently. This starts with the definition of appropriate goals (S1), continues with a consequent elaboration of requirements and specifications (S2), the implementation (S3), as well as an adequate verification and validation (S4), and ends with inadequate provision of tools for the operating/post-market phase (S5). The latter also relates to design transfer (P2) as well as governance principles. Even though basic responsibilities regarding governance are defined (G1), they remain vague and their implementation (G2) to (G4) is not pursued consequently. For example, this can be recognized by the missing analysis regarding the performance of the MDR. Additionally, the risks that the regulatory system itself poses are not actually analyzed/managed (G5) and systematic processes for reacting to issues are missing (G6).

Regarding general principles for product development and regulatory requirements in the field of medical devices, there often is a lack of transparency, clarity, and consistency (P1.a) compromising subsequent steps like a consequent and consistent implementation or validation of goals and requirements. Additionally, the MDR does not provide sufficient traceability across the development chain, violating principle (P1.b). There is no clear link between Recitals, Articles, and further components. In product development terminology, this means that there is no trace from goals and requirements to implementation steps as well as further activities like verification, validation, and post-market analysis. Based on this, the governance of the regulatory system remains limited. Major goals of the MDR cannot be rated adequately. For example, this applies to the practicability, efficiency, and flexibility of the regulatory system (P1.c) even though a “smooth functioning of the market” is defined by the MDR as a major goal. This also applies to the safety, performance, and effectiveness of the regulatory system, which is the second major objective (P1.d). At the end, a proper balance between these partially antagonistic goals cannot be systematically achieved, violating the principle of proportionality and impartiality (P1.e).

For example, this applies to the relationship between product safety on the one side and availability of medical devices for important treatment options on the other side. This also refers to a consequent implementation of a risk-based approach that properly adapts the amount of regulatory requirements to the actual risk of the device, e.g., regarding requirements for clinical evaluation and post market surveillance. Analogous to medical devices, a proper risk–benefit relationship needs to be achieved at the end. This has to take the risks of the regulatory system itself into account that it poses on the different actors, including manufacturers, healthcare institutions, authorities, and other organizations involved in regulatory processes. This needs to include a sufficient level of practicability and flexibility of the regulatory pathways and processes. Of course, it also needs to guarantee a sufficient level of product safety. However, this can only be judged when enough data about the functioning of the regulatory system is available and a comprehensive analysis regarding the performance of the regulatory system is provided. At the end, there should be a positive impact on the healthcare system itself, of which the medical device sector is a part.

On the one hand, the responsible actors in the regulatory system, like the EU commission and/or the MDCG, should pursue this. The MDR itself includes a central requirement in this regard. In Recital 85, it states: “The Commission should provide scientific, technical and corresponding logistical support to coordinating national authorities and ensure that the regulatory system for devices is effectively and uniformly implemented at Union level based on sound scientific evidence.” Thus, the EU should provide enough resources and adequate steps to realize this adequately. But, also other actors, like manufacturers and healthcare institutions as well as academia, should contribute. In general, the field of regulatory science should be better established in Europe. This field exactly addresses a systematic and scientifically valid analysis about the functioning of the regulatory system. Until now, the European institutions only provide limited resources and activities in this direction, as demonstrated in our analysis. Instead, the FDA in the United States defines regulatory sciences as an important field. It employs a substantial amount of regulatory scientists directly at the FDA and cooperates with a series of academic institutions in this field (24).

According to its own requirements, the MDR poses important requirements for transparency and adequate access to information. This does not only apply to medical devices but to the regulatory system in general. In particular, this is stated in Recital 43: “Transparency and adequate access to information, appropriately presented for the intended user, are essential in the public interest, to protect public health, to empower patients and healthcare professionals and to enable them to make informed decisions, to provide a sound basis for regulatory decision-making and to build confidence in the regulatory system.”

Currently, such goals are at most partially achieved. At the end, we have to conclude that the MDR does not meet its own standards, at the moment. If we apply the rules of the MDR to the MDR itself and the regulatory system built on it, it would fail and may not be placed on the market. Thus, improvements of the MDR and its regulatory system are recommended. Of course, regulations cannot be developed in the same way as medical devices. It may be argued, that regulations are not comparable to products and have distinct challenges regarding their development. For example, this is due to the complexity and variety of products that has to be managed by the regulation. Instead, a product/medical device has a more concisely defined focus and application context. However, central aspects and principles like (S1) to (S5) are cornerstones for the development of products for good reasons. Failure is likely when they are not consequently followed. These learnings should also be taken into account when developing a regulation. Basically, a regulation should be considered as a product that has to follow certain rules and standards in order to achieve proper functioning and the desired performance in its application context. According to its high impact on an entire industry sector and even more on the healthcare sector in general, medical device regulations should pursue high sophistication regarding a consequent application of standards. This is emphasized by the WHO document (18) that also establishes basic principles and rules for the development of regulations in the field of medical devices. Our approach extends these principles to a certain degree, since it more directly includes basic principles that are well known from product development.

Based on these considerations, our study elaborated basic deficiencies in the MDR. It demonstrates that improvements are not only possible but also indicated. In this regard, we appreciate current steps to pursue an update of the MDR. According to our analysis, it will be important that such an update is carefully designed to achieve actual improvements. Only when basic principles on regulatory systems as presented in this paper are systematically addressed, the MDR has a good chance to establish high-quality standards for itself.

In this paper, we only focused on the analysis of the MDR and its regulatory system. We did not include suggestions for future improvements, since this would have gone beyond our research question and could not be integrated on a solid scientific foundation. Such options were, e.g., discussed by trade unions and academic institutions in Huusko et al. (4), Nüssler (6), Kearney and McDermott (7), Biomedical Alliance in Europe (10), MedTech Europe (11), Shatrov and Blankart (14), Svempe (15), and Deutsche Industrie und Handelskammer, MedicalMountains, Spectaris (16). Further opportunities for improvements were provided in our statement within the public consultation regarding the targeted evaluation about the MDR and IVDR by the EU Commission, as presented in Haimerl et al. (25). This statement includes additional analysis steps that were not integrated into this paper. In general, this paper did not provide a comprehensive analysis of the entire MDR, but only addressed particular aspects as basic examples. In this study, the focus on prominent examples was utilized to showcase the deficiencies of the MDR. Additionally, a complete analysis would have exceeded the extent of this paper.

In the future, a more comprehensive analysis may be pursued to figure out the level of occurrence of such issues. This may be helpful to consequently approach potential issues in future versions of the MDR. Additionally, existing benchmarking tools for the evaluation of regulatory functions like the WHO Global Benchmarking Tool (26) or the Regulatory Impact Assessment from the OECD (27) could be utilized to assess the overall performance of the MDR in a comprehensive way. These tools basically focus on the set of functions that a regulatory system should contain and how they should be implemented. Additionally, they provide guidance regarding the evaluation of the regulatory systems and the processes used for this. The development process of the regulatory system is not a main focus of these benchmarking tools. Thus, this would have extended the basic scope of our study that was mainly directed towards the basic principles regarding the development of the regulatory system. But for certain aspects like the evaluation processes for the regulatory system as well as the comprehensiveness regarding the regulatory functions, the use of the benchmarking tools can be considered as a valuable extension for this study. Another option would be a comparative analysis in relation to medical device regulations in other areas or countries, e.g., the USA, Canada, Australia, or Japan. Based on this, it could be determined which of the regulations follows a clearly defined and balanced set of goals as well as the most consequent implementation of standards.

Another limitation of our paper is that we basically used the MDR for our analysis. To actually rate the level of performance of the MDR and its regulatory system, a detailed analysis of the relevant parameters would be necessary. This applies to parameters for assessing the levels of practicability, efficiency, and flexibility (P1.c) as well as the safety, performance, and effectiveness of the regulatory system. Only then, the principle of proportionality and impartiality (P1.e) and the corresponding success of the overall system can be rated. In this direction, some studies and position papers were published, e.g., by trade unions and academic institutions (4–7, 10, 11, 14–16). These are a good starting point to pursue a comprehensive analysis.

In summary, we have to state that the MDR would fail and could not be placed on the market when we would apply its own rules to the MDR and the regulatory system built on it. Even though regulatory systems do not need to match exactly the same standards on a 1:1 basis, our analysis demonstrates that the current status of the MDR contains several deficiencies regarding a consequent implementation with respect to standards for product development and regulatory systems. Our analysis also provided basic insights about important aspects that should be considered when developing and implementing a regulatory system like the one established by the MDR. This may help to properly address future improvements of the MDR. In particular, this is important because of the high impact the MDR has on all actors affected by the regulatory system, including manufacturers, healthcare organizations, patients, authorities, and other organizations interacting with it. At the end, the entire healthcare system and the society in general benefit from a regulatory system that achieves high quality in all relevant aspects and in the overall performance.

## Data Availability

The original contributions presented in the study are included in the article/supplementary material, further inquiries can be directed to the corresponding author.
